# Factors associated with extended length of stay for paediatric mental health presentations to EDs in South Western Sydney, Australia

**DOI:** 10.1111/1742-6723.70003

**Published:** 2025-02-11

**Authors:** Jahidur Rahman Khan, James Rufus John, Paul M. Middleton, Yao Huang, Ping‐I (Daniel) Lin, Nan Hu, Bin Jalaludin, Paul Chay, Raghu Lingam, Valsamma Eapen

**Affiliations:** ^1^ School of Clinical Medicine University of New South Wales Sydney New South Wales Australia; ^2^ Ingham Institute of Applied Medical Research Liverpool New South Wales Australia; ^3^ South Western Emergency Research Institute Liverpool New South Wales Australia; ^4^ School of Population Health University of New South Wales Sydney New South Wales Australia; ^5^ South Western Sydney Local Health District Liverpool New South Wales Australia

**Keywords:** ED, extended length of stay, mental health, paediatrics

## Abstract

**Objective:**

This study aimed to determine the factors associated with extended length of stay (LOS) for paediatric mental health (MH)‐related presentations to the EDs in South Western Sydney (SWS).

**Methods:**

We analysed electronic medical records (eMRs) of 7444 MH‐related ED encounters of children and young people (CYP) aged up to 18 years from all six public hospitals in SWS from January 2016 to April 2022. Extended LOS was defined as encounters of more than 4 h. We assessed factors associated with extended LOS using a multi‐level logistic regression model, accounting for hospital‐level clustering.

**Results:**

Approximately, 57.6% of all paediatric MH‐related ED presentations involved extended LOS. ED presentations by adolescents, patients with a culturally and linguistically diverse background, and those with ambulance arrival had increased odds of extended LOS compared to their counterparts. The odds of extended LOS were lower for encounters that occurred on weekends compared to weekdays, but the odds were higher for presentations that happened at night than during the day. Deliberate self‐harm, eating disorder, and schizophrenia spectrum disorders/psychosis‐related presentations had higher odds of extended LOS than other MH‐related presentations. Patients with MH presentations that required urgent evaluation (triage levels 1–2) had higher odds of extended LOS. Further, the odds of extended LOS were considerably lower during the COVID‐19 period compared to the pre‐COVID‐19 period.

**Conclusion:**

Our findings highlight the need for equitable distribution of resources directed towards at‐risk CYP to improve MH outcomes and reduce health system burden.

## Graphical abstract

Our analysis of 7,444 mental health‐related ED encounters of children and young people (CYP) aged upto 18 years from all six public hospitals in South Western Sydney showed that 58% had extended length of stay (LOS). Additionally, we found that ED presentationsby adolescents, patients with a culturally and linguistically diverse (CALD) background had increased odds of extended LOS compared to their counterpartswhereas the odds of extended LOS were considerably lower during the COVID‐19 period compared to the pre‐COVID‐19 period.

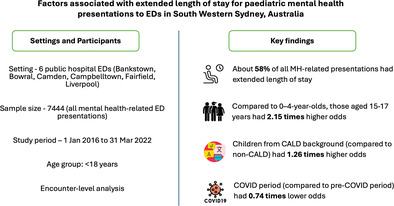

**Key findings**

- We found that more than one in two (57.6%) of all paediatric mental health (MH)‐related ED presentations involved extended length of stay (LOS).
- ED presentations by adolescents, those from a culturally and linguistically diverse background, ambulance arrivals, night‐time presentations, deliberate self‐harm, and priority triage (triage levels 1–2) were significantly associated with higher odds of extended LOS.
- The odds of extended LOS for paediatric MH‐related ED presentations were considerably lower during the COVID‐19 period compared to the pre‐COVID‐19 period.

## Introduction

Overcrowding in ED is a global issue reaching crisis proportions particularly since the COVID‐19 pandemic, resulting in both health and economic ramifications.^1^, ^2^ International evidence suggests that overcrowding in ED results in extended length of stay (LOS) at the ED, which in turn leads to adverse health outcomes, increased morbidity and mortality, and poor satisfaction among patients.^3^, ^4^ At health systems level, ED overcrowding is also associated with poor quality of care,^5^ dissatisfaction, and burnout among healthcare staff.^2^ Therefore, improving ED crowding by decreasing the LOS has a significant potential to not only improve health outcomes at patient‐level but also decrease the resource burden at the health systems level.

The Emergency Target Performance (formerly known as National Emergency Access Target [NEAT]) in Australia classifies a LOS of more than 4 h as extended.^6^ Several studies have reported that children and young people (CYP) who present to the ED with mental health (MH)‐related needs are likely to have an extended LOS.^7^, ^8^ Further, the recent COVID‐19 pandemic has significantly challenged the health systems in terms of increased demand for ED services,^9^ where our study showed a 16% increase in MH‐related ED presentations among CYP during the COVID‐19 period (March 2020 onwards).^10^ However, there is a scarcity of evidence on the paediatric ED LOS for MH presentations following the recent COVID‐19 pandemic.

Several factors, including sociodemographic, clinical, and hospital‐related characteristics, are linked to extended ED LOS. Previous studies in Australia have shown that factors such as patient age, mode of arrival, triage category, and reason for ED presentations were significantly associated with extended LOS.^11^, ^12^ However, these studies include all age groups including the elderly population, hence may have missed specific factors associated with extended LOS in ED among the paediatric population. Therefore, it is necessary to empirically identify factors associated with extended LOS in ED to mitigate such consequences.

Research into MH‐related ED use and LOS among Australia's culturally and linguistically diverse (CALD) populations, is limited in general. South Western Sydney Local Health District (SWSLHD) in NSW, is home to a substantial number of CALD residents compared to the rest of NSW (43% of the SWS residents are born overseas compared to the NSW state average of 34%). Social disadvantage (e.g. lower levels of educational attainment and income) is also notably high in this region compared to other parts of NSW,^13^ with a recent report featuring three suburbs in SWS in the list of top 10 communities severely impacted by COVID‐19 for children experiencing family employment stress.^14^ However, there is a dearth of research that attempts to ascertain the interplay between ED LOS and the sociocultural factors among CYP particularly in multicultural communities.

To address the above knowledge gaps, this study aimed to determine the impact of sociodemographic, sociocultural, treatment, hospital factors, and the COVID‐19 pandemic on extended LOS for paediatric MH presentations to ED.

## Methods

### Data source

We used electronic medical records (eMRs) to conduct a retrospective analysis of de‐identified records of paediatric MH‐related ED presentations between 1 January 2016 and 31 March 2022 among six public hospitals located in SWSLHD. The eMR data were securely accessed from the novel CEDRIC platform (Comprehensive Emergency Dataset for Research, Innovation, and Collaboration), which draws data from the Cerner PowerChart eMR system, and links all data points together to give a prospective, continually growing database of detailed patient journeys.^15^ CEDRIC is a unique automated research and clinical data linkage and analytical platform, utilising ‘big data’ pipelines and artificial intelligence (AI), to enable instantaneous data capture, communication, decision‐making and coordinated intervention. CEDRIC currently contains data on approximately 25 million hospital visits within the SWSLHD ED and hospital settings, but is being expanded to link seamlessly to databases outside the eMR. CEDRIC enables large‐scale data linkage for analytics in almost real time. CEDRIC currently runs on ‘on‐premises’ hardware, however the Big Data tools allow direct deployment into a cloud environment to create a hybrid ecosystem.^15^

### Cohort selection

ED encounters of CYP aged <18 years (at arrival) who presented for any MH problem during the 6‐year period were included. Principal diagnosis (ED discharge diagnosis) using International Classification of Diseases, 10th Division, Australian Modification (ICD‐10‐AM) codes was used to categorise presentations as MH‐related presentations. In the ED database, presenting problems were defined based on the Systematised Nomenclature of Medicine‐Clinical Terms Australian Extension codes (SNOMED CT‐AU), and we converted these codes into ICD‐10‐AM codes using the snoMAP‐Starter AU.^16^ A list of the ICD‐10‐AM codes used to categorise patients with MH disorders is shown in Table 1.

**TABLE 1:** ICD‐10 codes to define mental health conditions among children and adolescent

All mental health conditions	F codes (below) and all other codes specified here: E24.4, G31.2, G40.5, G62.1, G72.0, G72.1, I42.6 K29.2, K70, K85.2, K85.3, K86.0, O35.4, Y47, Y49, Z50.2, Z50.3, Z71.4, Z71.5, Z86.4, F55 G24.0, Z72.2, R78.1‐R78.5, Y87.0, Z91.5, X60‐X84, Y87.2, Y10‐Y34, Z86.5, Z09.3, Z50.4, Z91.4, R45.81.
Anxiety, obsessive‐compulsive disorders	F40–F42, F93.1, F93.2, F63.3
Autism spectrum disorder (ASD)	F84.0, F84.1, F84.5, F84.8, F84.9
Bipolar disorders*	F30, F31
Deliberate self‐harm (DSH)*	X60–X84, Y10–Y34, Y87.0, Y87.2, Z91.5, R45.81
Depressive disorders	F32–F39, F41.2, F25.1
Dissociative and conversion disorders	F44
Eating disorders	F50, excluding F50.5
Organic disorders	F00–F09
Personality disorders	F60, F61, F68.8, F69
Reaction and adjustment disorders	F43, F93.0, F94.0–F94.2
Schizophrenia spectrum disorders/psychosis*	F20–F29
Substance use‐related disorders	F10–F19, F55

* Age at presentation ≥10 years.

To ensure completeness of data using a conservative approach, we excluded ED encounters with duplicates and had missing data on sex, postcode, preferred language, country of birth, ED LOS, and any presentations by patients who left at their own risk. Since ED presentations, and not individuals, were analysed, multiple encounters by the same individual may have been included.

A list of outcome and predictor variables and their categories are presented in Table 2.

**TABLE 2:** Outcome and predictor variables

Variables and categories
**Outcome variable** Extended ED LOS (i.e. difference between time of arrival and time of departure) that was greater than 4 h as per the NEAT guidelines^6^ (≤4 h, >4 h).
**Predictor variables** Age at ED presentation (in years), gender (male/female), CALD status (yes/no), triage level (1, immediate; 2, emergency, within 10 min; 3, urgent, within 30 min; 4, semi‐urgent; within 60 min; 5, non‐urgent, within 120 min); deliberate self‐harm (DSH) (yes/no); day of arrival (weekday/weekend); time of arrival (00.00–07.59, 08.00–17.59 and 18.00–23.59); ambulance arrival (yes/no); residential area (postcode); socioeconomic status (SES); expressed using the Australian Bureau of Statistics (ABS) Census 2016 Index of Relative Socioeconomic Disadvantage [IRSD]) operationalised as quartile‐based categorical variables (least advantaged, second least advantaged, second most advantaged, most advantaged), area remoteness (major city, inner or outer regional), calculated based on the Area Remoteness Index of Australia (ARIA) classification, and COVID‐19 period (pre‐COVID‐19, i.e. January 2016 to February 2020; COVID‐19, i.e. March 2020 to March 2022). CALD status was determined if patients' preferred language was ‘non‐English’, or their birth country was a non‐English speaking country (excluding Australia, New Zealand, United Kingdom, Republic of Ireland, Canada, United States, or South Africa). In this study, triage categories 1 and 2 were grouped into one class, whereas categories 4 and 5 were grouped into one class (because of the low number of records in these categories) to indicate the severity of the condition, resulting in three triage classes (1–2, 3, 4–5). The SES index summarises a variety of socioeconomic factors (such as education, employment status, and income) of individuals and households within defined spatial units.^30^ Areas were divided into five classes according to the ARIA remoteness classification (major city, inner regional, outer regional, remote and very remote).^30^ Nevertheless, because the MH ED presentations in this study came from major city, inner regional, and outer regional areas, the remoteness variable was divided into two categories: ‘major city’ and ‘inner or outer regional’.

CALD, culturally and linguistically diverse; LOS, length of stay; MH, mental health; NEAT, National Emergency Access Target.

### Statistical analysis

We utilised descriptive statistics to make comparisons between the various groups of predictors and their respective rates of extended ED LOS. Following this, we utilised a multilevel logistic regression model to investigate the association between extended LOS and predictors, adjusting for Indigenous status and hospital level clustering. As presentations were clustered within hospitals, hospital‐level clustering was explored to evaluate hospital level variability. All analysis was performed using R version 3.6.3.

## Results

### Descriptive findings

This study analysed 7444 MH‐related ED encounters. The median LOS for MH ED presentation was 4.65 h (interquartile range [IQR] 5.41), with 57.6% of presentations exceeding the recommended threshold of 4 h, that is, extended LOS (Table 3). The median LOS for MH‐related ED presentations with an extended LOS was about 2.6 times that of presentations without an extended LOS (7.47 h *vs* 2.83 h). The highest share of all MH presentations was made up of females (63.0%) and adolescents (aged 12–17 years, 58.6%). The proportion of CYP from CALD population was 9.3%. Additionally, one in two (51.7%) presentations were made by CYP from socioeconomically deprived regions (i.e. Q1 least advantaged), with the majority coming from major cities in terms of remoteness. Approximately, a third (30.7%) of the MH‐related presentations were for DSH and 37.6% of all presentations were within the COVID‐19 period (Table 3). Among these presentations, 50.3% arrived by ambulance, 23.2% came to the ED on weekends, and 48% arrived after hours.

**TABLE 3:** Baseline characteristics of paediatric mental health presentations by extended ED LOS

	All presentations	Presentations without extended LOS (≤4 h)	Presentations with extended LOS (>4 h)
	*n* = 7444	*n* = 3155	*n* = 4289
LOS (hours), median (IQR)	4.65 (5.40)	2.83 (1.36)	7.47 (8.02)
Age at ED presentation (years)	*n* (%)	*n* (%)	*n* (%)
0–4	57 (0.8%)	42 (1.3%)	15 (0.3%)
5–8	165 (2.2%)	103 (3.3%)	62 (1.4%)
9–11	510 (6.9%)	280 (8.9%)	230 (5.4%)
12–14	2351 (31.6%)	1025 (32.5%)	1326 (30.9%)
15–17	4361 (58.6%)	1705 (54.0%)	2656 (61.9%)
Gender			
Male	2758 (37.0%)	1204 (38.2%)	1554 (36.2%)
Female	4686 (63.0%)	1951 (61.8%)	2735 (63.8%)
CALD			
No	6750 (90.7%)	2880 (91.3%)	3870 (90.2%)
Yes	694 (9.3%)	275 (8.7%)	419 (9.8%)
Ambulance arrival			
Yes	3747 (50.3%)	1781 (56.5%)	1966 (45.8%)
No	3697 (49.7%)	1374 (43.5%)	2323 (54.2%)
Day of arrival			
Weekday	5718 (76.8%)	2386 (75.6%)	3332 (77.7%)
Weekend	1726 (23.2%)	769 (24.4%)	957 (22.3%)
Time of arrival			
0–7.59	936 (12.6%)	257 (8.1%)	679 (15.8%)
8–17.59	3868 (52.0%)	1806 (57.2%)	2062 (48.1%)
18–23.59	2640 (35.5%)	1092 (34.6%)	1548 (36.1%)
Triage category			
Levels 1–2	642 (8.6%)	224 (7.1%)	418 (9.7%)
Level 3	4943 (66.4%)	1956 (62.0%)	2987 (69.6%)
Levels 4–5	1859 (25.0%)	975 (30.9%)	884 (20.6%)
Area socioeconomic status			
Least advantaged (1–25)	3847 (51.7%)	1583 (50.2%)	2264 (52.8%)
2nd least advantaged (26–50)	926 (12.4%)	352 (11.2%)	574 (13.4%)
Second most advantaged (51–75)	1165 (15.7%)	575 (18.2%)	590 (13.8%)
Most advantaged (76–100)	1506 (20.2%)	645 (20.4%)	861 (20.1%)
Area remoteness			
Major city	6488 (87.2%)	2632 (83.4%)	3856 (89.9%)
Inner or outer regional	956 (12.8%)	523 (16.6%)	433 (10.1%)
COVID period			
Pre‐COVID	4642 (62.4%)	1884 (59.7%)	2758 (64.3%)
COVID	2802 (37.6%)	1271 (40.3%)	1531 (35.7%)
Hospitals			
Bankstown	886 (11.9%)	378 (12.0%)	508 (11.8%)
Bowral	590 (7.9%)	447 (14.2%)	143 (3.3%)
Camden	66 (0.9%)	50 (1.6%)	16 (0.4%)
Campbelltown	3419 (45.9%)	1208 (38.3%)	2211 (51.6%)
Fairfield	201 (2.7%)	148 (4.7%)	53 (1.2%)
Liverpool	2282 (30.7%)	924 (29.3%)	1358 (31.7%)
Disease diagnosis groups			
Others*	1991 (26.7%)	831 (26.3%)	1160 (27.0%)
Anxiety, obsessive‐compulsive disorders	676 (9.1%)	448 (14.2%)	228 (5.3%)
Autism spectrum disorder (ASD)	34 (0.5%)	20 (0.6%)	14 (0.3%)
Bipolar disorders	21 (0.3%)	5 (0.2%)	16 (0.4%)
Deliberate self‐harm (DSH)	2289 (30.7%)	704 (22.3%)	1585 (37.0%)
Depressive disorders	725 (9.7%)	335 (10.6%)	390 (9.1%)
Dissociative and conversion disorders	59 (0.8%)	32 (1.0%)	27 (0.6%)
Eating disorders	48 (0.6%)	17 (0.5%)	31 (0.7%)
Organic disorders	201 (2.7%)	145 (4.6%)	56 (1.3%)
Personality disorders	548 (7.4%)	248 (7.9%)	300 (7.0%)
Reaction and adjustment disorders	295 (4.0%)	173 (5.5%)	122 (2.8%)
Schizophrenia spectrum disorders/psychosis	93 (1.2%)	15 (0.5%)	78 (1.8%)
Substance use‐related disorders	464 (6.2%)	182 (5.8%)	282 (6.6%)

CALD, culturally and linguistically diverse; LOS, length of stay; MH, mental health; NEAT, National Emergency Access Target.

* All other MH conditions.

### Findings of the multilevel logistic regression model

Table 4 summarises significant factors associated with extended LOS for paediatric MH ED presentations. Adolescents aged 15–17 years had significantly higher odds of having an extended LOS than children aged 0–4 years (adjusted odds ratio [AOR] 2.15, 95% CI 1.14–4.05). CYP with CALD background had higher odds of extended LOS than their counterparts (AOR 1.26, 95% CI 1.06–1.51).

**TABLE 4:** Factors associated with extended LOS in ED for mental health‐related conditions

Factors	AOR	95% CI	*P*‐value
Age at admission (years) (reference: 0–4 years)			
5–8	1.28	0.63–2.60	
9–11	1.39	0.72–2.68	
12–14	1.86	0.98–3.50	
15–17	2.15	1.14–4.05	*
Gender (reference: female)			
Male	1.04	0.93–1.15	
CALD (reference: no)			
Yes	1.26	1.06–1.51	*
Ambulance arrival (reference: no)			
Yes	1.17	1.05–1.30	**
Day of arrival (reference: weekday)			
Weekend	0.83	0.74–0.94	**
Time of arrival (reference: 8–17.59)			
00.00–07.59	2.43	2.04–2.89	***
18.00–23.59	1.27	1.14–1.41	***
Triage (reference: level 3)			
Levels 1–2	1.38	1.15–1.67	**
Levels 4–5	0.68	0.61–0.77	***
Area SES (reference: most advantaged [76–100])			
Least advantaged (1–25)	0.96	0.84–1.10	
Second least advantaged (26–50)	1.04	0.87–1.25	
Second most advantaged (51–75)	1.01	0.84–1.22	
Area remoteness (reference: major city)			
Inner or outer regional	1.14	0.92–1.41	
COVID‐19 period (reference: pre‐COVID)			
COVID	0.74	0.67–0.82	***
Disease diagnosis groups (reference: others†)			
Anxiety, obsessive‐compulsive disorders	0.48	0.40–0.59	***
Autism spectrum disorder (ASD)	0.7	0.34–1.46	
Bipolar disorders	2.78	0.97–8.01	
Deliberate self‐harm (DSH)	1.63	1.43–1.86	***
Depressive disorders	1.08	0.90–1.30	
Dissociative and conversion disorders	0.7	0.40–1.21	
Eating disorders	2.42	1.26–4.64	**
Organic disorders	0.42	0.30–0.60	***
Personality disorders	1.08	0.88–1.33	
Reaction and adjustment disorders	0.61	0.47–0.80	***
Schizophrenia spectrum disorders/psychosis	3.69	2.08–6.54	***
Substance use‐related disorders	1.07	0.85–1.36	
ICC_hospital_		0.12	

*P < 0.05; **P < 0.01, and ***P < 0.001. AOR, adjusted odds ratio; CI, confidence interval; ICC, intraclass correlation coefficient. †The “Others” category includes discharge diagnosis codes with a small number of records or records that do not fit into the above‐mentioned groups. Model adjusted for indigenous status as well as facility level clustering. Presentations with Indigenous status labelled as ‘Declined to respond’ and ‘Unknown’ were not included in this model.

Patients with MH‐related presentations that arrived in an ambulance had higher odds of having extended LOS than those that arrived via other means (AOR 1.17, 95% CI 1.05–1.30). The odds of an extended LOS were lower for presentations that occurred on weekends than during the weekdays (AOR 0.83, 95% CI 0.74–0.94), but the odds were higher for presentations that happened at night or late at night to early morning compared to those during the daytime (AOR 2.43, 95% CI 2.04–2.89 for 0–7.59; AOR 1.27, 95% CI 1.14–1.41 for 18.00–23.59). MH‐related presentations needing more urgent levels of care (triage levels 1 and 2) had higher odds of extended LOS than those with triage level 3 (AOR 1.38, 95% CI 1.15–1.67), whereas those with less urgent levels of care (triage levels 4 and 5) showed lower odds (AOR 0.68, 95% CI 0.61–0.77). When compared to presentations for other MH problems, presentations for DSH (AOR 1.77, 95% CI 1.59–1.98), eating disorder (AOR 2.42, 95% CI 1.26–4.64), and schizophrenia spectrum disorders/psychosis (AOR 3.69, 95% CI 2.08–6.54) had significantly higher odds of having an extended LOS. The odds of extended LOS were considerably lower during the COVID‐19 period compared to the pre‐COVID‐19 period (AOR 0.74, 95% CI 0.67–0.82). There was significant unexplained hospital‐level variation in the MH‐related extended LOS for CYP (intra‐class correlation coefficient = 12%).

## Discussion

This study reported on factors associated with extended LOS among CYP presenting to the ED with a primary MH diagnosis in a multicultural community in SWSLHD. During the 6‐year study period, 57.6% of MH‐related ED encounters had extended LOS. Additionally, the findings of this study also suggest that the likelihood of extended LOS for MH‐related ED presentations were higher for adolescents, those from a CALD population (e.g. non‐English‐speakers), ambulance arrivals, outside office hours, needing urgent levels of care (triage levels 1–2), and DSH‐related presentations. Additionally, we found that the odds of extended LOS were lower during the COVID‐19 period compared to the pre‐COVID‐19 period.

We found that about 58% of paediatric MH‐related ED presentations had extended LOS. This proportion is substantially higher than the nationwide increase in extended LOS for all ED presentations from 29% in 2017–2018 to 39% in 2021–2022.^17^ However, in line with other Australian studies,^18^, ^19^ CYP presenting to ED with MH problems were more likely to breach the NEAT timeframe than those with physical health problems. A study by Hiscock *et al*.^18^ showed that fewer MH presentations met the NEAT target than physical health presentations (65.4% *vs* 82.4%). Such increase may be compounded in SWSLHD because of the insufficient systems‐level resources available to meet the disproportionate increase in demand, given the large population growth in this area, particularly CYP (5.47% increase from 2016 to 2022 in 0‐ to 19‐year‐olds) as more young families are moving to this area because of affordability.^13^

We found several factors that were important predictors of extended LOS. Age was significantly associated with extended LOS where adolescents aged 15–17 years had the longest LOS compared to their younger counterparts. Consistent with other studies,^20^, ^21^ this may suggest that adolescents are more likely to have severe MH problems or complex psychosocial situations requiring more time for MH assessment and management. In terms of CALD status, we found that CYP whose preferred language was not English had extended LOS. This is consistent with the findings from other studies which might be explained by delays because of potential reasons of language barrier and the need for an interpreter, and in some cases, could be compounded by cultural barriers, for example, females from Middle Eastern cultures prefer to see female doctors.^22^

Patients who were brought to the ED by ambulance appeared to have a higher risk of having an extended LOS. This trend may suggest that patients who arrived at the ED via ambulance were more severely ill, necessitating longer treatment times.^21^ Further, patients who arrived outside office hours were more likely to have an extended LOS. One possible explanation is that there are fewer services and staff available, and may have had to wait for a longer time to be assessed and admitted or discharged.^23^ This study also found that weekend LOS were less likely to be extended. This may be because of the nature of MH conditions such that as a result of reduced access to primary care physicians on the weekend, CYP with less severe issues may be accessing the ED.^24^

Studies have reported that patients with less severe triage codes experienced longer waiting times compared to more severe cases that are usually prioritised.^25^, ^26^ Contrary to this, our study found that encounters that were triaged to urgent evaluation had extended LOS, perhaps needing more assessment and treatment time. Further, we found that 31% of all MH‐related ED encounters were DSH‐related, of which 37% had extended LOS. Such a high proportion of DSH‐related ED presentations has been reported in several other studies.^18^, ^21^ It is possible that most of the DSH‐related presentations would require an urgent triage code (Table S1), but it may take longer to organise MH specialist assessments in the ED, resulting in extended LOS. Moreover, such assessments may take more time because of reasons of needing both physical stabilisation based on the type of DSH (e.g. overdose needing treatment with antidotes and gastric lavage) as well as involvement of social care or child protective services, or delays related to involvement of parents/carers.^27^ Given the focus of this study, we only used the discharge status from the ED as the exit time for the LOS. Hence, we did not include admission or discharge status as a predictor of ED LOS because they are potential outcomes of ED LOS rather than predictors. This means that patients with an extended ED LOS are more likely to be admitted to the ward.

Despite the significant increase in MH presentations to ED during COVID‐19, we found that COVID‐19 period was associated with lower odds of extended LOS. This is consistent with a US study which reported that ED LOS was shorter during the COVID‐19 pandemic for patients with MH conditions discharged from the ED (10.3 min shorter) compared to the pre‐COVID period.^28^ It could be speculated that the EDs were less busy on the whole with allocation of ED capacity prioritised for the treatment of COVID‐19 cases, and patients presenting to ED with severe forms of MH problems were more likely to be transferred to MH units for assessment and/or admission rather than be managed in the ED itself, thereby these patients did not have extended LOS in the ED.^28^

### Limitations

While it is anticipated that administrative data are recorded with high accuracy within the eMR, the data are dependent on the accuracy and completeness of clinician documentation. Secondly, our study demonstrates an association rather than a causative relationship between predictors and outcomes. Thirdly, this study did not include any hospital‐level characteristics (e.g. staff numbers, paediatric beds), individual‐level sociodemographic variables (e.g. education, income), or spatial accessibility (e.g. distance to hospital) that may be associated with extended LOS. Finally, given the nature of the administrative data, details on the method of DSH such as cutting, self‐poising and so forth were not available which may have resulted in varied LOS. Future studies will need to take these into account when evaluating factors linked with extended LOS.

### Implications for policy and practice

Findings of this study show a disproportionate number of CYP with extended LOS which could be one of the reasons for significant overcrowding of EDs in SWSLHD. Future interventions including community‐based MH services, specialised psychiatric support for ED presentations, and re‐designing the physical ED environment to assess MH patients may reduce LOS. In line with this, NSW has implemented alternate care models such as Towards Zero Suicide and the ‘Safeguards’ Child and Adolescent Mental Health Rapid Response Teams, which are currently being implemented which may help redirect the patient load from EDs. Such programs are expected to provide targeted supports and thereby EDs to be less burdened with patient volume. Additionally, increasing capacity among general practitioners to manage MH problems at primary care level is also critical.

## Conclusion

Findings of this study show that these vulnerable subgroups who are at the highest risk for extended ED encounter need to be better managed. This reiterates the need to redirect resources to target improvements to not only the ED system but also the entire child and adolescent mental healthcare system. In this regard, an Integrated Continuum of Connect and Care (ICCC) model has been proposed that would integrate all relevant services using a tiered care pathway proportionate to the needs of the CYP and their families/carers.^29^ Directions for future research include ascertaining subjective experiences via qualitative interviews of children and adolescents and their families about the ED presentations, as well their subsequent service use including provision of appropriate care pathways. This might yield relevant information about the barriers and facilitators of service use and after care that will help shape equitable services for better management of acute paediatric MH presentations.

### Author contributions

JRK and JRJ conceptualised and designed the study, carried out the initial analyses, drafted the initial manuscript, and reviewed and revised the manuscript. PM is the Data Custodian of the CEDRIC platform. PM and YH contributed to data acquisition and results interpretation, and critically reviewed the manuscript. NH contributed to the methodology and diagnosis grouping. VE conceptualised and designed the study, coordinated, and supervised the study, and critically reviewed the manuscript. DL, BJ, PC and RL contributed substantially to results interpretation and critically edited the manuscript. All authors have approved the final manuscript as submitted and agreed to be accountable for all aspects of the work.

### Competing Interests

None declared.

## Supporting information


**Table S1.** Distribution of specific mental health conditions of ED presentation by Triage class.

## Data Availability

The data that support the findings of this study are available on request from the corresponding author. The data are not publicly available because of privacy or ethical restrictions.
