# Supplementary material for: Factors associated with extended length of stay for paediatric mental health presentations to EDs in South Western Sydney, Australia
Source: Emerg Med Australas. 2025 Feb 11;37(1):e70003. doi: 10.1111/1742-6723.70003 (PMC11811920; doi:10.1111/1742-6723.70003)
Supplement: Supplementary file 1 — Table S1. Distribution of specific mental health conditions of ED presentation by Triage class. [file EMM-37-0-s001.docx]

**Table S1. Distribution of specific mental health conditions of ED presentation by Triage class**

|  | **Triage** | | |
| --- | --- | --- | --- |
| **Disease diagnosis groups** | **1-2 (N=642)** | **3 (N=4943)** | **4-5 (N=1859)** |
| Anxiety, obsessive-compulsive disorders | 58 (8.6%) | 321 (47.5%) | 297 (43.9%) |
| Autism spectrum disorder (ASD) | 3 (8.8%) | 20 (58.8%) | 11 (32.4%) |
| Bipolar disorders | 0 (0.0%) | 16 (76.2%) | 5 (23.8%) |
| Deliberate self-harm (DSH) | 195 (8.5%) | 1663 (72.7%) | 431 (18.8%) |
| Depressive disorders | 22 (3.0%) | 478 (65.9%) | 225 (31.0%) |
| Dissociative and conversion disorders | 13 (22.0%) | 39 (66.1%) | 7 (11.9%) |
| Eating disorders | 2 (4.2%) | 20 (41.7%) | 26 (54.2%) |
| Organic disorders | 16 (8.0%) | 123 (61.2%) | 62 (30.8%) |
| Personality disorders | 45 (8.2%) | 369 (67.3%) | 134 (24.5%) |
| Reaction and adjustment disorders | 17 (5.8%) | 196 (66.4%) | 82 (27.8%) |
| Schizophrenia spectrum disorders/psychosis | 7 (7.5%) | 73 (78.5%) | 13 (14.0%) |
| Substance use related disorders | 128 (27.6%) | 225 (48.5%) | 111 (23.9%) |
| Others^¥^ | 136 (6.8%) | 1400 (70.3%) | 455 (22.9%) |
